# Telomere length: how the length makes a difference

**DOI:** 10.1007/s11033-020-05551-y

**Published:** 2020-09-02

**Authors:** M. Lulkiewicz, J. Bajsert, P. Kopczynski, W. Barczak, B. Rubis

**Affiliations:** 1 Department of Clinical Chemistry and Molecular Diagnostics, Poznan University of Medical Sciences, 49 Przybyszewskiego St., 60-355 Poznan, Poland; 2 Centre for Orthodontic Mini-Implants at the Department and Clinic of Maxillofacial Orthopedics and Orthodontics, Poznan University of Medical Sciences, 60-812 Poznan, Poland; 3 Department of Head and Neck Surgery, Poznan University of Medical Sciences, The Greater Poland Cancer Centre, 61-866 Poznan, Poland; 4 Radiobiology Laboratory, Department of Medical Physics, The Greater Poland Cancer Centre, 61-866 Poznan, Poland

**Keywords:** Telomeres, Telomerase, Immortality, Aging, Sex, Hormones

## Abstract

Telomerase is perceived as an immortality enzyme that might provide longevity to cells and whole organisms. Importantly, it is generally inactive in most somatic cells of healthy, adult men. Consequently, its substrates, i.e. telomeres, get shorter in most human cells with time. Noteworthy, cell life limitation due to telomere attrition during cell divisions, may not be as bad as it looks since longer cell life means longer exposition to harmful factors. Consequently, telomere length (attrition rate) becomes a factor that is responsible for inducing the signaling that leads to the elimination of cells that lived long enough to acquire severe damage. It seems that telomere length that depends on many different factors (including telomerase activity but also genetic factors, a hormonal profile that reflects sex, etc.) might become a useful marker of aging and exposition to stress. Thus in the current paper, we review the factors that affect telomere length in human cells focusing on sex that all together with different environmental and hormonal regulations as well as parental aspect affect telomere attrition rate. We also raise some limitations in the assessment of telomere length that hinders a trustworthy meta-analysis that might lead to acknowledgment of the real value of this parameter.

## Introduction

Telomere dynamics is associated with aging and senescence. Both terms often appear together, but they describe a bit different set of metabolic changes. Aging is the attenuation of physiological functions that comes with time and, eventually, leads to cell death. Primary drivers of aging are telomere damage, epigenetic dysregulation, DNA damage, and mitochondrial dysfunction. Noteworthy, aging is the major risk factor for cancer, cardiovascular disease, diabetes, and neurodegenerative disorders as well as chronic obstructive pulmonary disease, chronic kidney disease, osteoporosis, sarcopenia, stroke and many others [1]. Consequently, some of these drivers can induce senescence that is the process of stable, irreversible growth arrest of cells. Apart from evident changes in the phenotype of an organism, senescence also causes chromatin remodeling, metabolic changes, increased autophagy, and release of numerous proinflammatory factors [2]. Senescence can occur due to aging or due to age-related diseases and can be induced by therapeutic agents (e.g. chemotherapeutic drugs). It plays roles in normal development, maintains tissue homeostasis, and limits tumor progression. Noteworthy, the primary role of senescence (an irreversible state) is to limit the proliferation of damaged cells that prevents potential malignant cell transformation [3]. However, it also has been implicated as a major cause of age-related diseases. In mammalian cells, two types of senescence may be listed, i.e., (i) intrinsic senescence—induced by telomere attrition or dysfunction and (ii) extrinsic senescence—caused by external signals (from the outside of the cell or by signals that are telomere-independent) [4]. The most critical and widely recognized senescence markers are considered β-galactosidase/SAβ-gal, p16 INK4A, histone modifications, and microRNAs profile alteration as well as genomic instability and mitochondrial dysfunction that may reflect exposition to stress [5, 6]. Additionally, a pro-inflammatory secretome (cytokines and matrix metalloproteinases) are also recognized as the senescence-associated secretory phenotype that may also contribute to aging [7, 8]. Significantly, most of those hallmarks are regarded as characteristics of a cancer cell [6]. Interestingly, a strong relationship between short telomeres and mortality risk, particularly at younger ages, was suggested but without further clinical implications so far [9].

We are still looking for aging markers that might be controlled so we could prevent or slow down this phenomenon. Importantly, age is one of the critical risk factors in cancer development that is associated with the increased exposition time to potentially harmful compounds and processes (including stress). Thus, it is crucial to be able to monitor both aging and senescence by using specific markers that would provide a diagnostic as well as predictive value. It seems that telomere length (TL) might be a good candidate for such a marker. However, due to large variability, huge dynamics, and assessment issues, the value of this parameter still seems to show a limited accuracy.

## Telomeres in stress

Telomeres protect the chromosomes’ integrity. They get shorter with every cell division in most cells since telomerase is not active in the vast majority of human cells [10]. One might say that they “sacrifice" themselves. Especially since losing a telomeric sequence is not as critical for the current metabolic status of a cell as losing the sequence of DNA coding for a protein. Other functions of telomeres include preventing chromosomes from joining or being recognized as DNA double-strand breaks, which, in turn, would alert DNA repair mechanisms [11]. However, over time, telomere length in normal cells decreases until they become too short to play their role and keep cells capable of dividing. This results in cellular senescence that is one of the major causes of aging and aging-related disorders [12] associated with the end replication problem (Hayflick limit) [13].

Telomerase expression and activity in human cells decrease with age [14]. However, it is an individual phenomenon and the telomere attrition rate is associated, among others, with the ability to conquer stress. As suggested, physical activity (perceived as one of the main ways to attenuate stress level) is associated with healthy aging and reduced risk for several chronic disorders, possibly due to telomere restoration [15, 16]. At the cellular level, stress refers to the factors that can affect the metabolism or can damage the cell. However, regardless of the level, stress accumulates progressively along with aging, and it may negatively affect the individual’s health and well-being. Importantly, antioxidants were shown to delay the onset of vascular senescence in a telomerase dependent way. In an in vitro study, the reactive oxygen species (ROS) were shown to decrease the level of nuclear hTERT (human telomerase reverse transcriptase) protein (the key telomerase subunit) and telomerase activity in endothelial cells, which was followed by a senescent phenotype development. At the same time, incubation with the antioxidant N-acetylcysteine blocked this nuclear export of hTERT into the cytosol, suggesting its role in stress response [17]. Another well-known antioxidant—a-Tocopherol, was shown to repress telomere shortening and retain telomerase activity in brain microvascular endotheliocytes [18]. Similarly, Ginko Biloba extract (of high antioxidant potential) was shown to delay the onset of senescence through activating telomerase via PI3k/Akt signaling pathway [19, 20]. Importantly, chronic stress results in an increased secretion of cortisol that is capable of suppressing telomerase activation in the immune system and, consequently, promoting telomere attrition [21].

The fact is that aging is accompanied by telomere attrition, although its rate is highly heterogeneous between individuals, different types of cells but also different chromosomes [22]. Especially the latest aspect leads to the conclusion that cells can undergo senescence prematurely, even when the average telomere length is "normal," but some specific chromosome ends are critically short. Since there are some biochemical pathways common to aging, stress response, and telomere attrition, we do believe that chromosome ends are very sensitive stress markers and reliable indicators of cellular aging. However, it seems that the only way to acknowledge telomere length as a senescence/aging/exposition-to-stress marker is to assess the length/attrition-rate of individual single chromosomes and not measuring the total telomere length on average.

Another crucial report was demonstrated by Garrett-Bakelman et al., who demonstrated a study associated with the exposition to decreased gravity. As shown, an astronaut (Scott Kelly), who spent almost a year on the international space station, experienced some severe syndromes (reduced body mass, instability in his genome, swelling in major blood vessels, changes in eye shape, metabolism shifts, inflammation and alterations in his microbiome) but also a significant telomere lengthening. Importantly, the chromosome ends shortened again after the astronaut landed and returned to nearly preflight levels within 6 months after return to Earth. However, the increased number of short telomeres were observed, and the expression of some genes was still disrupted. At the same time, his identical twin brother (did not spend the time in space) was monitored as a reference showing no significant alterations in telomere length [23]. This case shows how complex telomere regulation is.

## ALT in telomere homeostasis and aging

It was reported that in some cases, telomeres could be restored even without the telomerase activity. This phenomenon is known as an Alternative Lengthening of Telomeres (ALT) and is based on the homologous recombination of telomeric DNA. As revealed, it is supposed to be present in 15–20% of tumors lacking active telomerase and was demonstrated to sufficiently overcome the replicative senescence in mammalian somatic cells in vitro [24]. There are also some reports showing both mechanisms coexisting in the same cells, but it was suggested to result from the experimental design rather than to be a common phenomenon [25]. Interestingly, ALT was also demonstrated in normal mouse somatic tissues [26]. It may be that this not very common mechanism is just turned on in some particular conditions or cell types. It is difficult to tell how this rescue system is controlled and if we could use it for aging delay or monitoring. Since this seems to be a marginal system and refers to cancer cells mainly, it may not constitute a base for a promising perspective in the context of modulation of telomere attrition, aging, or senescence.

## (Re)activation of telomerase

It seems reasonable that aging symptoms could be limited while applying telomerase activators. Such factors would safely restore telomere length or stimulate cell resistance to apoptosis induced by oxidative stress and would eventually attenuate senescence [27]. The ability to modify telomerase activity was already reported [28–32]. Some reports show that aging could be reverted by telomerase reactivation not only in a mouse model [29] but also in human cells [30]. Moreover, it was shown that normal physiological aging could be delayed without increasing the incidence of cancer, as demonstrated in adult wild-type mice (pharmacological activation or systemic viral transduction of telomerase) [31, 32]. As shown in an old rat model, telomerase induction mediated by irisin (a secreted myokine) was accompanied by autophagy induction and improved mitochondrial function in aged hepatocytes [33]. Another telomerase activator, TA-65 (extracted from *Astragalus Membranaceus*) that is capable of increasing average telomere length and decreasing the percentage of critically short telomeres, was studied in a mouse model. As demonstrated, it significantly improved particular well-being indicators, including glucose tolerance, osteoporosis markers, and skin fitness, without significantly increasing global cancer incidence [31]. It was also shown to improve the metabolic activity of mouse cells as well as better dynamics of the immune system in humans. Similarly, another telomerase activator, AGS-499, was demonstrated to play neuroprotective effects in mice [30, 34]. Similarly, the same compound was shown to protect human bone marrow mesenchymal stem cells (hMSC) from apoptosis and DNA damage induced by H2O2, and from the toxicity caused by long term exposure to DMSO. Consequently, some regenerative potential of telomerase inducers was suggested [35]**.** It was also demonstrated that L-carnitine could improve the aging-related symptoms due to increasing telomerase activity, decreasing aging, and changing the methylation status of hTERT promoter [36]. Similar results were shown in the study of MSCs isolated from the adipose tissue and an antioxidant, ZnSO_4_. The compound contributed to slower aging due to lengthening of telomeres, increasing *hTERT* expression and telomerase activity. Simultaneously, *hTERT* promoter methylation was altered [37]. Alternatively, a major component of cigarettes, cotinine, was shown to activate telomerase causing abnormal proliferation [38]. In addition, it was also reported that activation of telomerase could reactivate the proliferative capability of benign tumors [39]. Some other reports show that telomerase activity modulation could contribute to the control of cell migration, differentiation, senescence or the cell cycle (DNAzymes used as modulators) showing some non-canonical functions of telomerase and telomeres [40]. Consequently, some concerns about the safety of telomerase restoration are raised and should be verified.

## Telomere length profiling

It must be emphasized that telomere length is not equally distributed among individual chromosomes. Thus it is crucial to be aware that some of the methods would benefit only as screening methods but very accurate. The methods for telomere length assessment were revised elsewhere with all the advantages and disadvantages discussed thoroughly [41]. Briefly, there are two basic approaches used in telomere length assessment, i.e. (i) evaluation of the average length of telomeres and (ii) evaluation of the range of individual telomeres in respective chromosomes. The first group of methods gives a general idea about the telomere attrition while the second one is more precise that results from the fact that the shortening of individual chromosomes may be different and the shortest telomere is critical for cell viability and chromosome stability [42]. The first group of the methods (average length assessment) covers southern blot (more precisely TRF—terminal restriction fragment) and qPCR (with some modifications). The length of individual chromosomes endings may be assessed with the use of more sophisticated methods (and their variants) like Q-FISH (quantitative fluorescent in situ hybridization), Flow-FISH or STELA (single telomere elongation length analysis) [43]. They do differ in equipment and handling time requirements, but they also do give some different quality results. Consequently, it is challenging to compare results from different laboratories that use diverse methods. They do correspond to each other relatively but not quantitatively. Thus, it gives an idea about the telomere length alterations (shortening rate in fact) but not about the real telomere length.

In conclusion, association studies of TL and human health or disease suffer from some methodological issues, including lack of reproducibility and diversity in handling methods. Importantly, telomere length, as a very dynamic parameter, can be altered by some environmental stimuli comprising hormonal profile fluctuations or therapeutic interventions. Thus, it is essential to integrate clinical data in the molecular studies of telomere length that would help in the evaluation of the factor as a significant disease(s) biomarker.

## Telomere assessment and clinical outcome—high hopes

An inverse relationship between age and telomere length is commonly known. Similarly, it is known that telomere length in leukocytes (LTL) is positively correlated with a number of years of disease-free living [44, 45], which may suggest leukocyte telomere length as a biomarker for healthy aging.

Since telomere length is perceived as a potential marker of cell metabolism, medical condition, resistance to stress, etc., it became a good candidate for a solid predictive and/or diagnostic marker. The issue is that telomere attrition rate, although known as an average 20–50 nucleotides per year [46], is, first of all, not so easy to assess. As mentioned above, different methods give different results. Secondly, telomere length that we are born with also varies, and the range is quite wide, i.e., 8 to 15 Kb [47]. Noteworthy, placenta-derived material is a perfect source for the assessment of the primary newborns’ telomere length that enables further monitoring. Alternatively, telomere length assessment can be based on the analysis of liquid biopsy samples (blood cells), which makes it relatively easy to perform. Furthermore, as several studies showed, measurement of telomere length in placenta and blood has a great clinical value.

In the study monitoring telomere length from birth to adulthood, an inverse correlation between residential traffic exposure and telomere length in the first two decades of life was shown [48]. Additionally, a correlation between the effects of prenatal exposures and maternal conditions such as obstetric complications, BMI, state of nutrition, stress and sociologic status during pregnancy, and disadvantageous birth outcomes of offspring in telomere length manner was shown [49–51]. For example, the association between maternal stress during pregnancy and shorter offspring telomere length was confirmed by many authors [52–54]. Other authors revealed an association between low birth weight and shorter telomere length in peripheral blood mononuclear cells in preschool aged children [55]. In another study, maternal exposure or perinatal complications was linked to shorter leucocytes telomere length in adulthood with some accelerated aging symptoms [56]. Also, the exposition to stress during pregnancy or early childhood was associated with shorter telomere length 70 years later but (survivors of the siege of Leningrad). Interestingly, the authors noticed no direct effect on the prevalence of cardiovascular diseases [57].

When assessed from cells in the bloodstream, telomeres can show variation from their genetically predisposed lengths due to environmental-induced changes. These alterations in telomere length act as indicators of cellular health, which, in turn, could provide disease risk status [58]. The common goal of such study is to assess alterations in telomere length rather than absolute values. Importantly, even if the attrition rate may differ between individuals, it usually leads to a value of fewer than 4-kilo bases in old age [59]. Since it is a dynamic process, all the comparative studies must include the difference in age and sex (will be discussed further) of the individuals.

Concerning the usefulness of telomere length assessment and its clinical outcome, it must be emphasized that those studies are still more descriptive than mechanistic. Their length shows some correlations with various disorders [60] including obesity but also lower socioeconomic position, smoking, mortality, or sex [61–66]. Telomere metabolism and function are complex, and it may be challenging to obtain a full picture of the correlation. It seems that all those studies give just a general perspective on the postulated association, but considering the current state of knowledge on telomere biology, it may be very constructive.

## How sex makes a difference—telomere length in women and men

The variations in TRF length among newborns are as wide as variations in TRF lengths among adults [47]. Additionally, a faster telomere attrition appears in men than in women [67]. However, there is no consistency in the evaluation of the association between sex and telomere length in adults, and this hypothesis has evolved dynamically. Some studies show that white blood cell telomeres are longer in women than men [66, 68, 69]. It could result from the presence of an estrogen response element in the hTERT promoter region, which may affect the expression of this gene and contribute to telomere restoration [69, 70].

Noteworthy, the telomeric sequences (i.e., G-reach repeats) are particularly sensitive to oxidative stress [71]. Importantly, women show lower reactive oxygen species (ROS) than men due to higher levels of estrogen [69, 72]. This hormone reduces the production of ROS while also being a potent antioxidant and regulator of antioxidant genes [73]. It can also directly activate the hTERT promoter [74] and indirectly affect DNA repair (p53-mediated pathway) [75] as well as activate telomerase through the phosphoinositol-3-kinase⁄Akt [76] and nitric oxide pathways [77]. These mechanisms could result in females having higher telomerase activity. However, since telomerase is generally perceived absent in most somatic adult cells, it is difficult to evaluate how it affects telomere length in different tissues.

Conversely, testosterone does not show antioxidant properties but is associated with increased susceptibility to oxidative stress that may lead to telomere attrition [78]. Importantly, androgens are converted to estrogens in the presence of aromatase [79]. However, even untreated postmenopausal women show slower leukocyte telomere attrition than age-matched men [70]. Thus it seems that estrogen-related control of telomerase activation is crucial but not the only one that contributes to this process. Altogether, a huge diversity of theories and reported results may come from differences in the age of study subjects, small study groups, different cell types subjected to DNA isolation, or different assessment methods. One of the most informative and wide meta-analyses was reported by Gardner et al., who analyzed 36,230 cases and showed that, on average, females had longer telomeres than males. Importantly, the difference significance was prone to assessment method with only Southern blot, but neither real-time PCR nor Flow-FISH showing a significant difference [65]. Interestingly, an experiment concerning dizygotic twins confirmed that observation. It was demonstrated that female‐derived leukocyte telomeres were generally longer than in the male twin [80]. Additionally, girls show a stronger relationship between exposure to air pollution and telomere shortening [81], which shows another aspect of the complex regulation of telomerase and telomeres.

## Mother effect

Since telomere length seems to be a useful stress marker, it can be expected that telomere length in the newborn is associated with maternal exposures during pregnancy. This dependency was observed among male newborns, not females. Shorter telomere length was linked to risk factors as smoking during pregnancy, high mother's BMI, maternal depressive state, and sexual abuse [82]. Another association between telomere length in mother and newborn is mediated by the hypothalamic–pituitary–adrenal axis during pregnancy. There is no association along with maternal cortisol level and telomere length of the newborn. However, it was noticed that cortisol levels in third trimesters were higher at mothers of males infants than females. Presumably, this phenomenon affects telomere attrition rate depending on sex, and precisely, male offspring seem to be influenced predominantly [83].

It may be that this results from the fact that maternal hormones (naturally occurring or released in response to a stress etc.) during pregnancy are associated with epigenetic modifications of the fetal glucocorticoid receptor coding gene, NR3C1. Noteworthy, male and female fetuses develop different responses to adapt to this exposure [83]. But except for only some cases, the general observations indicate equal telomere length in both sex newborns.

## Father effect

It was demonstrated that the age of the father at conception was positively correlated with a longer leukocyte telomere length in newborns. This, in turn, is associated with a reduced risk of atherosclerosis and longer, healthy life (noteworthy, it also correlates with a higher risk of achondroplasia, Marfan syndrome, as well as neurodevelopmental disorders, e.g., autism) [84]. These observations were confirmed in studies accompanied by sperm telomere length assessment using Southern blot, Q-FISH, and flow-FISH [85]. Interestingly, the correlation was linear, indicating a father's age a crucial factor in the determination of newborns' LTL. It may be associated with the mechanisms that affect telomere elongation in sperm, but the detailed mechanism is not known yet. Importantly, this correlation appears even in spite of the "reprogramming" during embryonic development [86–88].

## Summary

The studies of the molecular basis of aging and telomere length suggest that there may be some important factors that do affect the inborn telomere length but also the rate of chromosome end attrition. An important issue is prenatal exposure to environmental conditions but also the hormonal profile and age of parents. It may be that pollution, increased inflammation processes, and oxidative stress in adulthood may indeed accelerate telomere length attrition. However, the association between parents’ and newborns’ telomere length seems to be a critical factor as well. We should not forget that telomere length reflects the entire life history of the individual from birth onward. Importantly, early childhood is associated with higher metabolic turnover, and consequently, faster shortening of telomeres [89]. It must be noted that telomere metabolism is a very dynamic process that is controlled by genetic as well as environmental determinants and is very individual. Even if it seems too simplified, it may be that slowing down telomere attrition could result in postponed senescence and aging. Alternatively, the induction of telomerase in healthy patients might also bring similar results. We are still not sure about the potential of telomerase activity, and the role of individual subunits of the complex, especially since some important non-canonical functions are postulated when hTERT is reported. It may also be that telomere length could become o good predictive or diagnostic marker in the assessment of the human's health condition, but till that time, we need first to learn how telomeres are metabolized and how to reliably assess this parameter. On the other hand, telomerase and telomeres are still recognized as good cancer therapy targets, which only shows how complex is the whole signaling network. There is a concern raised when telomerase restoration is considered that is associated with the safety precautions since the enzyme may lead to the immortality of cancer cells. On the other hand, it is well established that physical activity slows down telomere attrition, and it is generally perceived as a good manifestation of a healthy lifestyle. But other factors must be included in those studies like exercise intensity and BMI that may significantly affect the observations.

To conclude, we still need to learn how telomerase and telomeres work. We also need to work on some new diagnostic methods that would enable telomere assessment over life, to become good predictive, diagnostic markers.
